# High pathogenicity avian influenza (HPAI) H7N6 virus detected in New Zealand poultry

**DOI:** 10.1128/mra.00088-25

**Published:** 2025-04-29

**Authors:** Michelle McCulley, Andrew David Wilson, Ruy Jauregui, Edna Gias, Yee Syuen Low, Sylvia Ohneiser, Wlodek Stanislawek, Loren Bryant, Anastasia Chernyavtseva, Joseph O'Keefe

**Affiliations:** 1 Animal Health Laboratory, Diagnostics, Readiness and Surveillance, Biosecurity New Zealand, Ministry for Primary Industries, Upper Hutt, New Zealand; Katholieke Universiteit Leuven, Leuven, Belgium

**Keywords:** HPAI, H7N6, avian viruses, New Zealand, poultry, genome

## Abstract

We report the genome sequence of a high pathogenicity avian influenza (HPAI) virus (*Alphainfluenzavirus, Orthomyxoviridae*), subtyped as H7N6, sampled on a poultry farm November 2024 in the Otago region of New Zealand’s South Island. The genome was sequenced with Oxford Nanopore sequencing technology.

## ANNOUNCEMENT

Avian influenza viruses (genus *Alphainfluenzavirus,* family *Orthomyxoviridae*) are pathogens of worldwide concern in public health. Following a mortality event in free-range poultry in Otago, in New Zealand’s South Island, clinical swabs and tissue samples from domestic *Gallus gallus* were tested at the Animal Health Laboratory, Ministry for Primary Industries, Wellington, New Zealand for high pathogenicity avian influenza (HPAI). Total nucleic acid was extracted from 31 oropharyngeal swabs and four pooled tissue samples (*n* = 35) using the MagMAX core nucleic acid purification kit (Applied Biosystems, USA) on a KingFisher Flex system (Thermo Fisher Scientific, USA).

Of the 35 samples, 33 (29 swabs, four pooled tissue) tested positive for Influenza A by qRT-PCR (1), while two were invalid. Additionally, 17 samples (13 swabs, four pooled tissue) were positive for subtype H7 (2). Among the remaining samples, 15 had inconclusive H7 qRT-PCR results (Ct >40), and three were invalid. From the 33 Influenza A-positive samples, those with Ct <30 (*n* = 18) were selected for whole genome amplification by RT-PCR using previously published methods and primers (3). Amplicons were purified with ExoSAP-IT (Thermo Fisher Scientific) and quantified using the Qubit 1x dsDNA high sensitivity assay (Thermo Fisher Scientific).

All 18 samples were prepared using the Rapid Barcoding Kit 96 V14 (SQK-RBK114.96, Oxford Nanopore Technologies, UK), pooled, then sequenced on a P2 Solo device (Oxford Nanopore Technologies). Basecalling was performed using Dorado (v7.0.2) with a high accuracy basecalling model (HAC DNA v4.3.0). Raw reads were trimmed using BBDuk v39.01 before mapping to a non-redundant reference segment set downloaded from GISAID using Minimap2 v2.24, followed by generation of consensus sequences. Full genomes, comprised of eight segments each, were able to be assembled from 14 of the 18 samples; the remaining four samples were excluded due to poor quality assemblies. All genomes were subtyped as H7N6 via BLAST analysis of H and N sequences. Due to high genomic similarity, a representative genome (A/Chicken/New Zealand/W24_2595/2024) was selected for further analysis. Full genome assembly statistics for the representative genome is presented in Table 1.

**TABLE 1:** Genome assembly statistics, closest NCBI BLAST hits, GISAID accessions, and GenBank accessions for each H7N6 HPAI viral genome segment

Genome assembly statistics							
BioProject accession			PRJNA1195966				
Raw read number			731,101				
Number of mapped reads			221,820 (30.3%)				
N50 (Kb)			0.580				
GISAID accession			EPI_ISL_19587280				

Segment	Length (bp)	Number of mapped reads		Average depth of coverage	Closest NCBI blastp match (Accession, pairwise ID)	GISAID accession	GenBank accession
1 - PB2	2,280	12,648		3,034×	ADF45466.1 (New Zealand H7N7), 98.4%	EPI3671862	PQ675393.1
2 - PB1	2,277	6,387		1,217×	ADF45464.1 (New Zealand H7N7), 99.6%	EPI3671863	PQ675394.1
3 - PA	2,151	21,918		4,019×	XHB94090.1 (New Zealand, H1N9), 99.3%	EPI3671864	PQ675395.1
4 - HA	1,689	1,842		560×	AWG44370.1 (New Zealand H7N7), 95.6%	EPI3671865	PQ675396.1
5 - NP	1,497	26,427		8,681×	AHZ37569.1 (Sweden, H10N7), 99.4%	EPI3671866	PQ675397.1
6 - NA	1,413	23,607		6,369×	AHZ13549.1 (New Zealand, H4N6), 97.7%	EPI3671867	PQ675398.1
7 - MP	948	49,093		17,907×	Multiple accessions, 100%	EPI3671868	PQ675399.1
8 - NS	693	79,898		20,640×	QNQ32092.1 (USA, H4N6), 98.3%	EPI3671869	PQ675400.1

**Fig 1:**
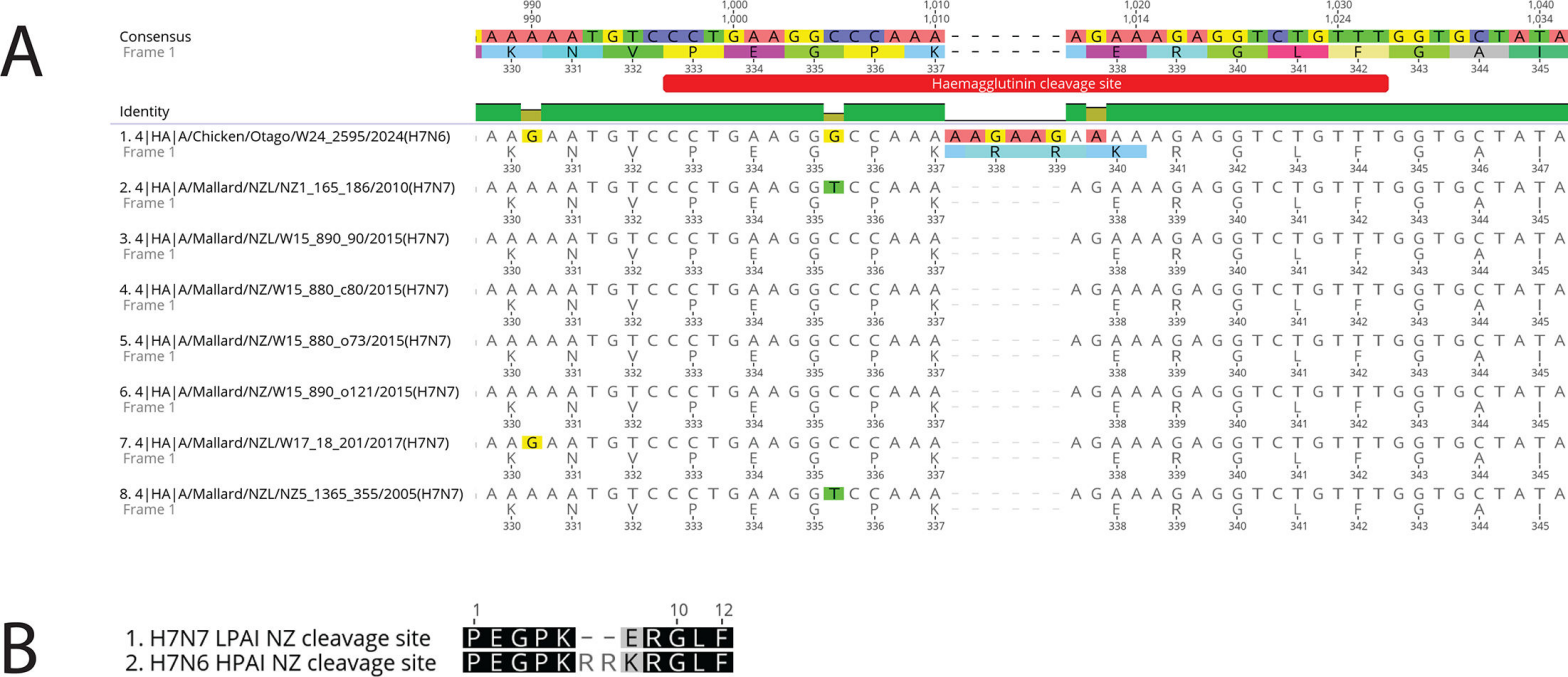
*In silico* detection of multi-basic cleavage site, indicating evolution from LPAI to HPAI. (**A**) Nucleotide alignment illustrating a six-nucleotide insertion event and a single nucleotide variant affecting the cleavage site of the hemagglutinin (HA) gene. (**B**) Amino acid alignment showing the differences between New Zealand LPAI and HPAI H7 cleavage sites. Alignment figures produced using Geneious Prime v2021.1.1 (https://www.geneious.com).

All segments were translated and analyzed using BLAST (Table 1), showing strong similarity to endemic New Zealand H7N7 and H4N6 LPAIs (4). Seven H7N7 genome sequences from an internally curated LPAI database were available for comparison. Alignment of HA segments using MAFFT v7.490 revealed a six-nucleotide insertion and a separate single nucleotide variant within the H7N6 genome, both affecting the HA cleavage site (Fig. 1A). These mutations increased the number of basic amino acids from two to five, enabling characterization as HPAI based on the Offlu cleavage site analysis guide (https://www.offlu.org) (Fig. 1B). Given the similarity to endemic LPAI H7N7 genomes (Table 1), these data suggest an evolutionary transition from endemic LPAI to HPAI as a consequence of (a) reassortment event(s).

All protocols and software were used per manufacturer instructions unless stated otherwise.

## Data Availability

The sequenced genome has been deposited in GISAID under the reference number EPI_ISL_19587280 and in the NCBI with accessions listed in Table 1. In addition, raw data are available under the SRA accession number SRR31649752. Primer sequences were removed prior to upload.
